# High-k Three-Phase Epoxy/K_1.6_(Ni_0.8_Ti_7.2_)O_16_/CNT Composites with Synergetic Effect

**DOI:** 10.3390/polym14030448

**Published:** 2022-01-22

**Authors:** Maria Vikulova, Tatyana Nikityuk, Denis Artyukhov, Alexey Tsyganov, Alexey Bainyashev, Igor Burmistrov, Nikolay Gorshkov

**Affiliations:** 1 Department of Chemistry and Technology of Materials, Yuri Gagarin State Technical University of Saratov, 77 Polytecnicheskaya Street, 410054 Saratov, Russia; vikulovama@yandex.ru (M.V.); tanya.saratov19@gmail.com (T.N.); mr.tokve@gmail.com (D.A.); tsyganov.a.93@mail.ru (A.T.); ambal281191@gmail.com (A.B.); 2 Engineering Center, Plekhanov Russian University of Economics, 36 Stremyanny Lane, 117997 Moscow, Russia; burmistrov.in@rea.ru

**Keywords:** three-phase composites, epoxy resin, hollandite, carbon nanotubes, dielectric properties, synergism

## Abstract

Polymer matrix composites based on ED-20 epoxy resin, hollandite K_1.6_(Ni_0.8_Ti_7.2_)O_16_ and carbon nanotubes with a variable content of 0.107; 0.213 and 0.425 vol.% were obtained for the first time. Initial components and composites produced were characterized by XRD, XRA, FTIR, SEM and Raman spectroscopy. The dielectric properties of composite materials were measured by impedance spectroscopy and determined by the volume ratio of the composite components, primarily by the concentration of CNTs. At a CNT content of 0.213 vol.% (before percolation threshold), the maximum synergistic effect of carbon and ceramic fillers on the dielectric properties of a composite based on the epoxy resin was found. Three-phase composites based on epoxy resin, with a maximum permittivity at a minimum dielectric loss tangent, are promising materials for elements of an electronic component base.

## 1. Introduction

Polymer matrix composites attract great attention in the scientific community and industry due to the wide possibilities for regulating their properties (dielectric, mechanical, etc.), depending on the potential field of application, by varying the qualitative and quantitative composition of the composite material, which consists of choosing the type of matrix and filler, as well as their mass/volume ratio.

When choosing a polymer matrix, as a rule, the choice is based on its flexibility/hardness (determined by the intended area of use), a technologically simple process for obtaining composites, as well as its acceptable dielectric parameters, primarily, a low dielectric loss value.

The type of filler, which can have a dielectric or conductive nature, is justified by its electrophysical characteristics, primarily high values of permittivity, and good dispersibility in the corresponding polymer.

Currently, two-phase composites are widely used. Ceramic fillers for polymer matrices are simple and complex oxides of various compositions and structures [1,2,3,4,5,6,7,8,9]. Common conductive additives for two-phase composites are a variety of metals [10,11,12,13,14] and carbon materials [15,16,17,18,19]. The advantages of such composites are predictable dielectric properties, relatively low dielectric losses, and ease of manufacture [20,21]. However, significant problems in their use are associated with the deterioration of the mechanical and technological properties, since an increase in permittivity is achieved near the percolation threshold at high concentrations of hard ceramic particles in the polymer matrix.

Hence, in recent years, three-phase composite materials have gained the greatest popularity. In this case, by partially replacing ceramic particles with conductive ones it is possible to produce polymer matrix composites with a sharp increase in permittivity near the percolation threshold of conductive particles. This effect was achieved using highly conductive fillers in the form of metals and carbon nanotubes [12,14,22,23,24,25].

Epoxy resin, as one of the materials commonly used in the field of electronics and electrical engineering, has insulating properties, resistance to thermal decomposition and chemical stability. The best results were reported for epoxy systems doped with silver flakes with a permittivity of 2000 [26]. However, such a noticeable increase in permittivity is always accompanied by a significant increase in electrical conductivity and dielectric losses due to the presence of an insulator–conductor interface. In this case, high sensitivity of the permittivity value to the content of conductive fillers is observed. A small deviation from the percolation threshold can lead to a serious drop in permittivity, which makes it difficult to control the parameters of the composite preparation process.

Dielectric properties of two-phase composites based on epoxy resin with ceramic or carbon filler are characterized by the permittivity from 100 to 200 at 1.5 vol.% of MWCNT depending on frequency (10^2^–10^6^ Hz) [27] and 5.5–6.0 at 0.2 vol.% BaTiO_3_ as the most studied ceramic material (10^−1^–10^6^ Hz) [28].

The production of three-phase composites based on the epoxy resin is not widespread. It is known that epoxy resin with hybrid carbon filler based on graphene nanoplates and multi-walled nanotubes has high thermal conductivity [29]. MWCNT/TiO_2_-epoxy nanocomposite demonstrates good mechanical properties under various stresses [30].

Studies of dielectric properties of epoxy three-phase composites with simultaneous use of ceramic and carbon fillers are sporadic. From recent research, hybrid composites based on epoxy with fixed MWCNT content above (0.09 vol.%) and below (0.58 vol.%) percolation threshold and varied MnFe_2_O_4_ up to 10 vol.% demonstrated a permittivity of up to 100 and 1000, respectively, at optimal MnFe_2_O_4_ concentration, room temperature and 129 Hz [31].

Within the framework of this work, it is planned to create polymer matrix composites with an expected synergetic effect based on epoxy resin and a filler in the form of a complex oxide K_1.6_(Ni_0.8_Ti_7.2_)O_16_ with a hollandite-like structure and with a conductive addition of carbon nanotubes for the first time.

The efficiency of hollandite-like structures in the creation of polymer-matrix composites with an optimal combination of dielectric properties was previously shown using the example of systems [32,33,34]. Carbon nanotubes (CNTs) are known for their excellent conductivity and, at a relatively low content, do not deteriorate the mechanical properties of composites, which together opens up wide opportunities for the development of new composite polymer matrix materials.

The aim of this work is to synthesize and study composite materials based on epoxy resin, a ceramic material with a hollandite-like structure, and carbon nanotubes.

## 2. Materials and Methods

Ceramic material of K_1.6_(Ni_0.8_Ti_7.2_)O_16_ (KNTO) with hollandite-like structure was synthesized by technique, described in the research [35,36].

To obtain polymer matrix composites, powders of a ceramic filler and carbon nanotubes (CNTs) (Taunit-M, OOO NanoTechCentre, Tambov, Russia) were pre-mixed to produce ceramic-CNT composite with the subsequent introduction into ED-20 epoxy resin (GOST 10587-93, CHS-Epoxy 520, Usti nad Labem, Czech Republic). The hardener is triethylenetetramine (TETA, TU 6-02-1099-83). The qualitative characteristics of ED-20 and TETA as the hardener are presented in Table 1.

**Figure 1:**
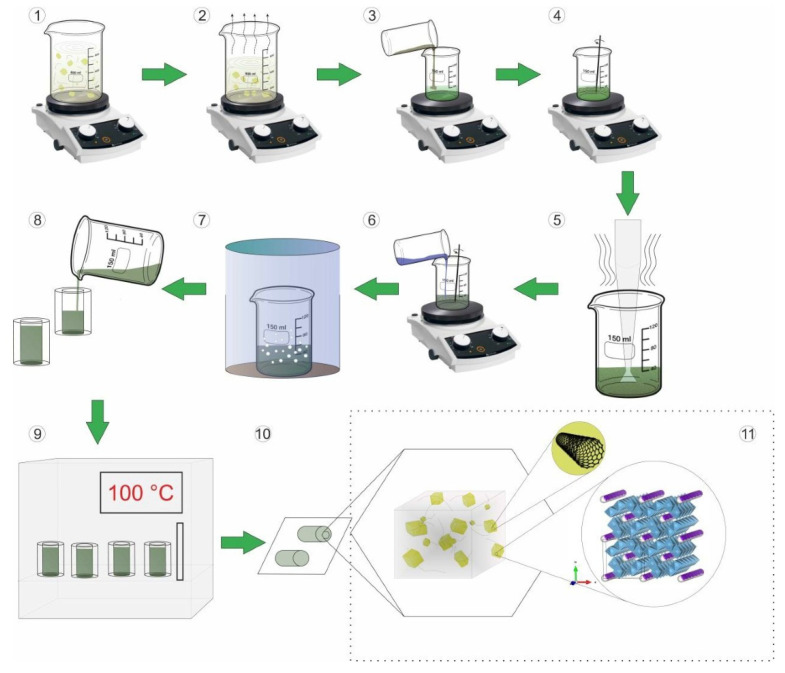
Scheme of three-phase composites based on ED-20, KNTO and CNTs.

Ceramic-CNT composites with fixed KNTO content and different content of CNTs were prepared by mixing of components dispersion in ethanol, stabilized using ultrasonic treatment for 1 h (step 1 Figure 1). The solvent was evaporated with constant stirring while heating on a magnetic stirrer and followed by drying in an oven at 100 °C (step 2 Figure 1). Obtained ceramic-CNT composites were added into ED-20 epoxy resin (step 3 Figure 1) and homogenized with magnetic stirrer (15 min, step 4 Figure 1) and ultrasonic homogenizer (1 h, step 5 Figure 1). Then, the hardener triethylenetetramine (TETA, TU 6-02-1099-83) (about 15% of the ED-20 mass) was added to the homogeneous mixture and stirred for several minutes (step 6 Figure 1). The mixture was degassed under vacuum at 25 ± 5 °C for 30 min (step 7 Figure 1). The resulting homogeneous mixture was poured into a cylindrical shape with a diameter of 11 mm (step 8 Figure 1), left to cure at room temperature, and then in an oven at 100 ± 5 °C for 2 h (step 9 Figure 1). Then, the samples were removed from the mold (step 10 Figure 1), and tablets with a diameter of 11 mm were obtained using cut-off machine Accutom-5 from Struers (Copenhagen, Denmark), and polished on a grinding machine Tegramin-20 from Struers to a thickness of 1.5 mm. A schematic representation of the obtained three-phase composites based on epoxy resin, KNTO and CNTs is shown in area 11 in Figure 1.

To compare the dielectric characteristics, two-phase composites based on epoxy resin and carbon nanotubes were also obtained. The content of carbon nanotubes was 0.107, 0.213 and 0.425 vol.% (CNTs density of 1.9 g/cm^3^ was used to recalculate mass fraction to volume fraction [37]), the content of K_1.6_(Ni_0.8_Ti_7.2_)O_16_ towards epoxy-CNT was 20.1, 19.5, 18.9 vol.%. The used composite components and obtained composites are designated short names (Table 2 and Table 3).

The particle size distribution of a complex oxide with a hollandite-like structure was obtained using an ANALYSETTE 22 MicroTec plus laser particle size analyzer from FRITSCH (Idar-Oberstein, Germany). X-ray phase analysis of a complex oxide with a hollandite-like structure was obtained using diffractometer ARL X’TRA Thermo Scientific (Reinach, Switzerland). Morphology and elemental composition of hollandite, as well as the distribution of fillers and structural features of polymer matrix composites, was investigated using a scanning electron microscope ASPEX Explorer with annex for the energy dispersive X-ray analysis (Framingham, USA). Fourier-transform infrared spectroscopy (FTIR) was carried out using FTIR spectrometer FT-801 (Novosibirsk, Russia). Raman spectroscopy was carried out using NTEGRA Spectra NT-MDT (Amsterdam, Netherlands).

The dielectric characteristics of composite materials based on epoxy resin were investigated using a Novocontrol Alpha AN impedance meter (Montabaur, Germany). The measurements were carried out in the frequency range from 100 Hz to 1 MHz at room temperature with a measuring signal of 1 V. To prepare the samples for examination, conductive glue «Kontaktol» (Keller) was applied to them on both sides, followed by drying at room temperature for 24 h.

## 3. Results and Discussion

An increase in permittivity in three-phase composites is achieved, first of all, due to the introduction of a ceramic filler, the function of which in the system under study is performed by a complex oxide of the composition K_1.6_(Ni_0.8_Ti_7.2_)O_16_ with a hollandite-like structure. Polarization processes in such materials are caused by the mobility of K^+^ ions in quasi-one-dimensional channels of the tunnel structure, accompanied by a redistribution of electrons in the structural lattice due to the variable valence of titanium and nickel in the hollandite composition [36].

Single-phase ceramics with a hollandite-like structure based on nickel potassium titanate with comparable dielectric properties with hollandites of different chemical compositions are characterized by simplicity and a variety of synthesis methods, including a solid-phase reaction [38] and citrate–nitrate modification of the sol-gel technology [39,40], as well as the approach of thermal treatment of an X-ray amorphous precursor, potassium polytitanate, chemically modified in an aqueous solution of a nickel salt, which is also used within the framework of this work.

The typical morphology of particles of hollandite-like materials is columnar, which complicates charge transfer and necessitates the use of conducting particles, which are chosen as carbon nanotubes.

**Figure 2:**
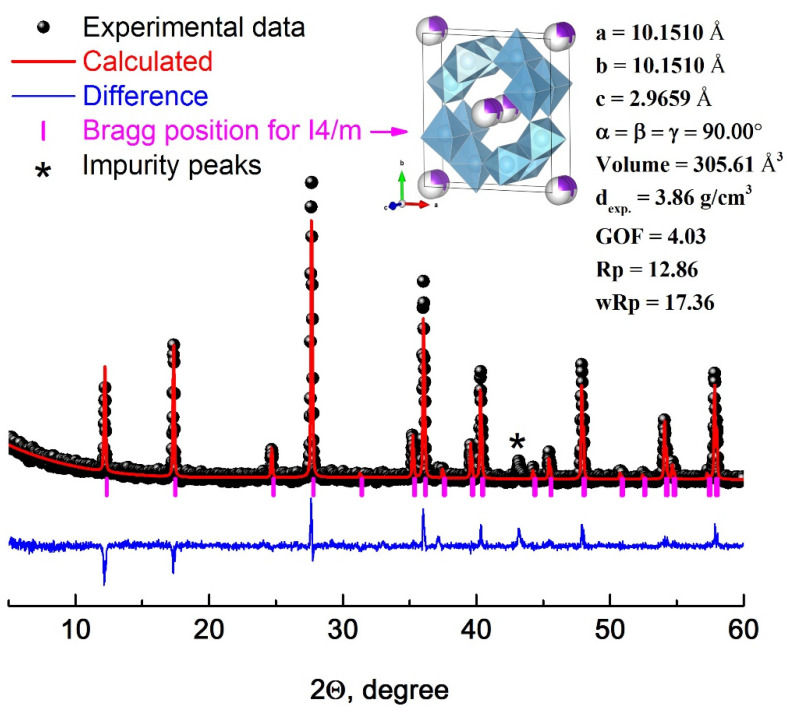
X-ray diffraction pattern of K_1.6_(Ni_0.8_Ti_7.2_)O_16_ with structure refinement by the Rietveld method.

The structure of the obtained complex oxide was confirmed by X-ray phase analysis (Figure 2).

The diffraction pattern identifies a single crystalline phase of a complex oxide with a hollandite-like structure, characterized by the space group I4/m. To refine the parameters of the crystal lattice of the obtained ceramic filler, the Rietveld method was used. The Figure shows the difference (blue line) of the experimental (black balls) and calculated (red line) diffraction patterns. A slight discrepancy is observed in the case of some reflexes with the highest intensity (profile factor Rp = 12.86%, weighted profile factor wRp = 17.36%). The structural parameters of the unit cell were determined: a = 10.1510 Å, b = 10.1510 Å, c = 2.9659 Å, α = β = γ = 90°, the unit cell volume is 305.61 Å^3^, the theoretical density is 3.86 g/cm^3^. The goodness of fit (GOF = 4.03) is rather low and indicates a high level of agreement between theoretical and experimental data. An elementary cell constructed from the obtained data is also shown in Figure 2.

**Figure 3:**
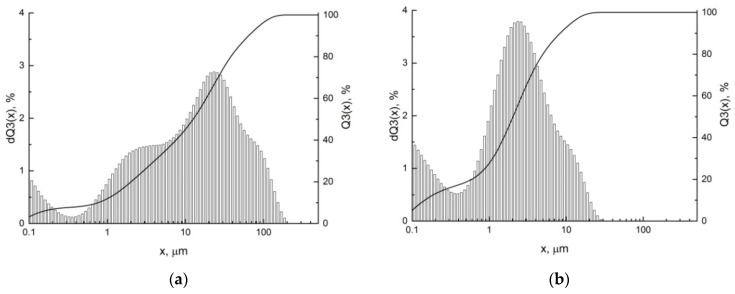
The particle size distribution of ceramic filler K_1.6_(Ni_0.8_Ti_7.2_)O_16_ with hollandite-like structure after synthesis (**a**) and after additional treatment during ceramic-CNT composite producing (**b**).

The synthesized ceramic filler in the form of a complex oxide of the composition K_1.6_(Ni_0.8_Ti_7.2_)O_16_ with a hollandite-like structure was studied by the method of laser diffraction after synthesis (Figure 3a) and after additional treatment during ceramic-CNT composite production (Figure 3b). According to the differential histogram, after synthesis the ceramic powder consists of three fractions: nanosized particles (<0.1 μm), small agglomerates (~2 μm) and larger aggregates (~20 μm) of particles, while the fraction of the particles smaller than 1 μm is approximately 14 vol.%, which is established by the integral curve (Figure 3a). Consequently, obtained ceramic material consists of nanosized particles prone to agglomeration. This is confirmed by the result that after additional treatment the fraction of the particles smaller than 1 μm increases up to 28 vol.%. The average particle size is reduced to 2 μm (Figure 3b).

**Figure 4:**
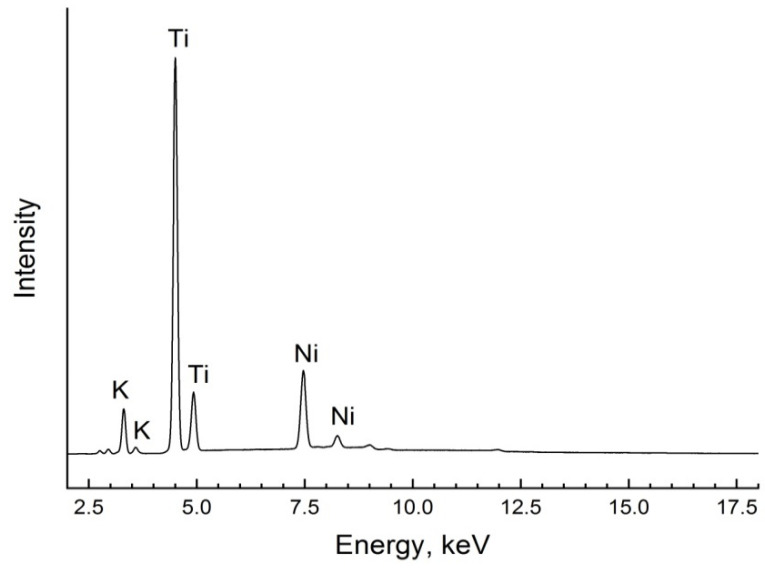
EDX spectra of ceramic filler K_1.6_(Ni_0.8_Ti_7.2_)O_16_ with hollandite-like structure.

The presence of structure-based chemical elements in the composition of the ceramics synthesized was confirmed using energy dispersive X-ray analysis (Figure 4).

The EDX measurements show the contents of K, Ti and Ni in synthesized ceramics with a hollandite-like structure. Consequently, the introduction of nickel in the structure of potassium titanate has happened.

**Figure 5:**
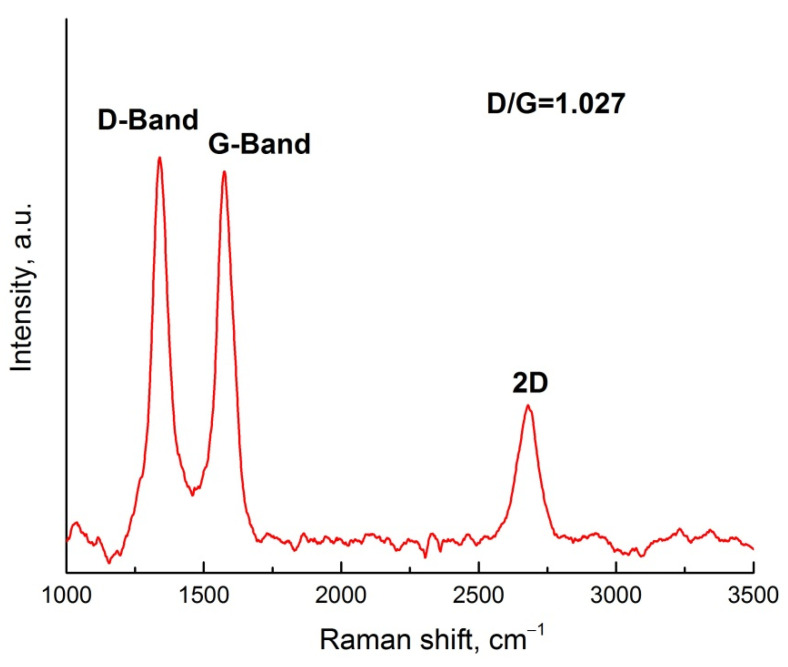
Raman spectra of carbon filler CNT.

Raman spectrum confirms the typical characteristics of carbon nanotubes, which were used as carbon filler in three-phase composites (Figure 5).

There are three peaks at 1360 cm^−1^, 1600 cm^−1^ and 2700 cm^−1^, which is traditionally called the D-band, G-band and 2D peak. The presence of impurities or disorder in the carbon-based structure is indicated by the D-peak. The G-peak appears as a result of carbon–carbon bond stretching. The 2D peak is common in most of the carbon samples and its width, intensity and location give information about the number of sample layers. The ratio of intensities of the D-band and G-band is calculated and has a value of 1.027.

**Figure 6:**
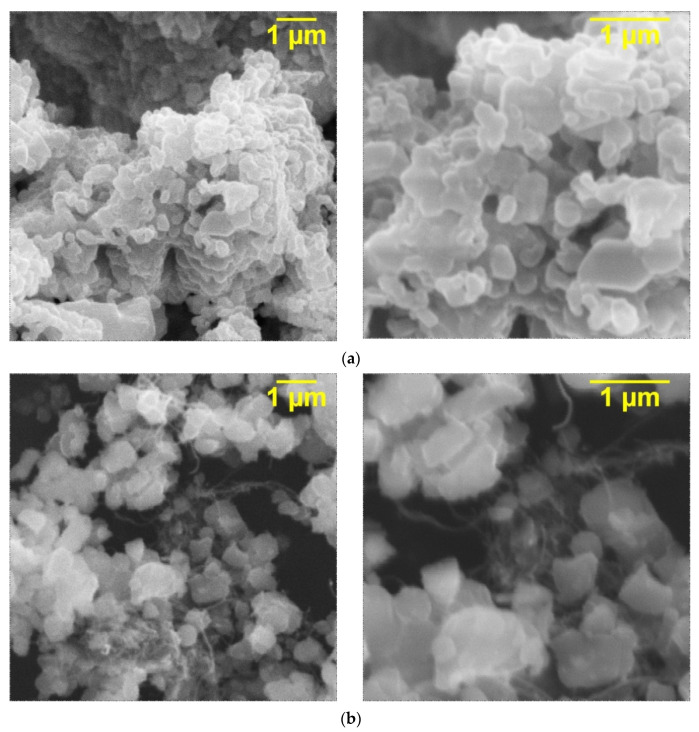
Electron micrographs of (**a**) initial ceramic filler K_1.6_(Ni_0.8_Ti_7.2_)O_16_ and (**b**) ceramic-CNT composite.

Ceramic-CNT composites based on KNTO and carbon nanotubes were obtained before adding to the epoxy resin to produce three-phase composites. Their electron microphotographs in comparison to the initial ceramics are shown in Figure 6.

Hollandite-like material, after synthesis and before additional treatment for ceramic-CNT composite production, has irregularly shaped particles that form agglomerates with a size of 10 microns or more. From Figure 6b it can be seen that the size of the KNTO particles in the ceramic-CNT composite composition decreased. Additionally, carbon nanotubes around KNTO particles are clearly detectable on SEM.

**Figure 7:**
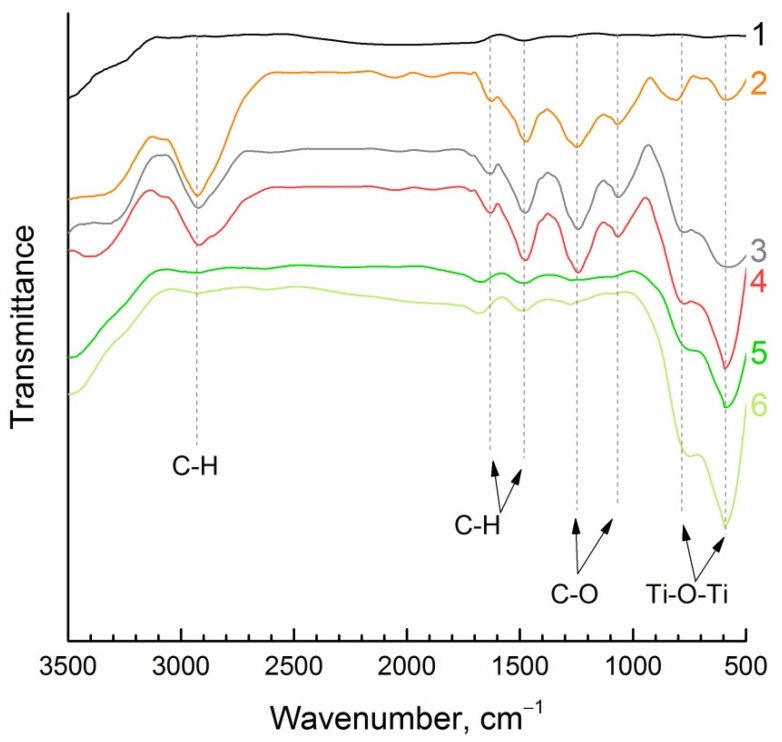
FTIR spectra of (1) carbon filler CNT, (2) initial polymer matrix, composites with different composition (3) (Epoxy-CNT)-KNTO; (4) Epoxy-KNTO; (5) KNTO-CNT and (6) ceramic filler KNTO.

FTIR spectrum analysis was employed to characterize the raw materials and intermediate composites as well as analyze the changes in the structure of polymer and fillers during composite production. The FTIR spectra for the initial epoxy resin and composites with different compositions are shown in Figure 7.

The FTIR spectra of the pure polymer matrix and polymer matrix composites have adsorption bands of functional groups that are typical for epoxy resin: C-H band at 2935 cm^−1^ (methoxyl groups); C-H band at 1605 cm^−1^ and 1500 cm^−1^ (aromatic ring); C-O band at 1230 cm^−1^ and 1100 cm^−1^ (aromatic ring). Additionally, characterization evidence of the epoxy resin was observed in the bands of oxirane ring at ~800 cm^−1^ and ~600 cm^−1^. Ceramic filler has typical Ti–O–Ti bands at ~750 cm^−1^ and ~600 cm^−1^ in the FTIR spectrum. The FTIR spectrum of carbon filler does not have adsorption bands of functional groups. It can be seen that new functional groups did not appear and existing ones did not disappear. That is why initial composite components save their structure and do not have a destructive effect on each other.

**Figure 8:**
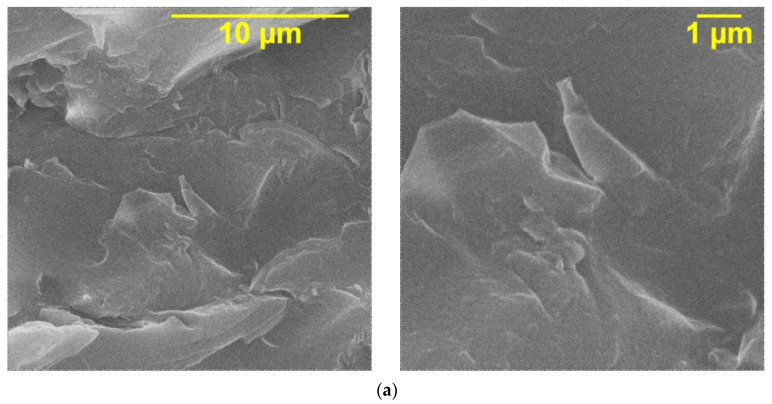

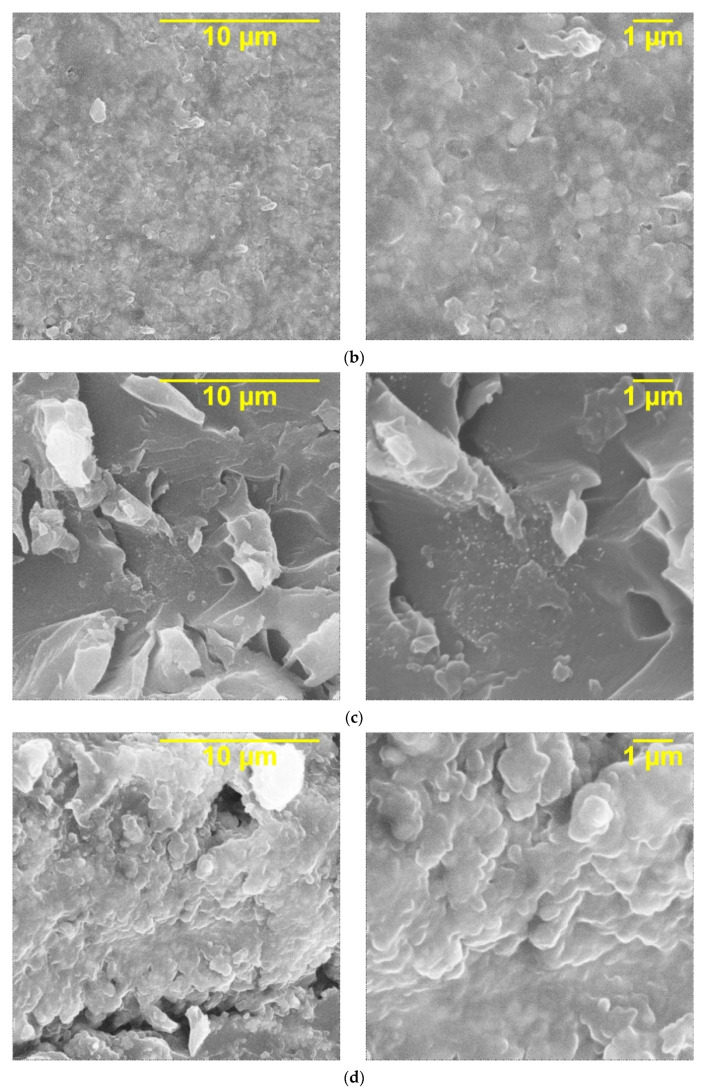
Electron micrographs of (**a**) initial epoxy resin and polymer matrix composites with compositions: (**b**) Epoxy-KNTO; (**c**) Epoxy-CNT; (**d**) (Epoxy-CNT)-KNTO.

The structure of the initial epoxy resin, assessed by an electron micrograph of the cleavage (Figure 8a), is characterized by a large number of voids, which noticeably deteriorates the properties of the resulting composite.

However, a comparison of the samples shows that when the epoxy resin is mixed with carbon nanotubes (Figure 8c), no voids were found. In this case, an inhomogeneous, highly developed cleavage surface is observed.

Additionally, it was found that in the absence of CNTs, hollandite particles are poorly distributed in the polymer matrix and form rather large agglomerates (Figure 8b). As can be seen from Figure 8d, the addition of CNTs has a positive effect on the distribution of ceramic filler particles.

**Figure 9:**
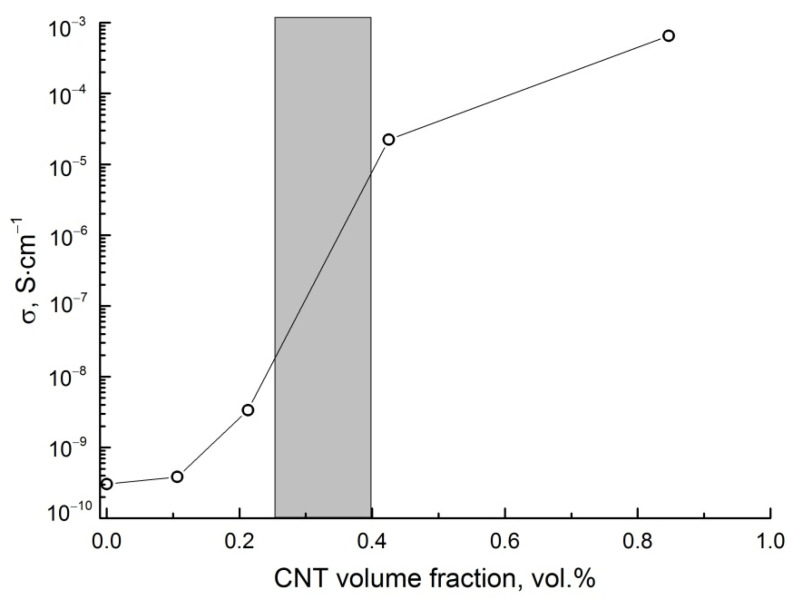
The conductivity of composites versus CNT volume fraction at 50 Hz.

It should be noted that the CNT content for composite production was selected before and after the percolation threshold, where a sharp increase in the conductivity was observed at low frequency (*f* = 50 Hz) suggesting the formation of a tunneling conductive network. This area is grayed out in Figure 9.

**Figure 10:**
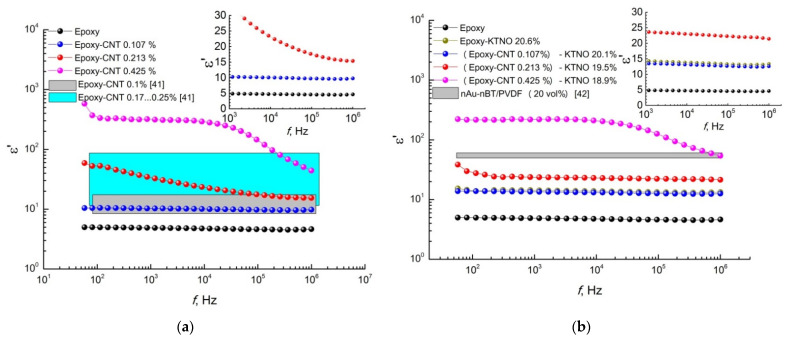
The frequency dependence of permittivity (**a**) two- and (**b**) three-phase composites compared to pure epoxy resin. Color areas in this Figure: (**a**) numerical simulation for similar two-phase composites [41], (**b**) experimental data for similar three-phase composite [42].

**Figure 11:**
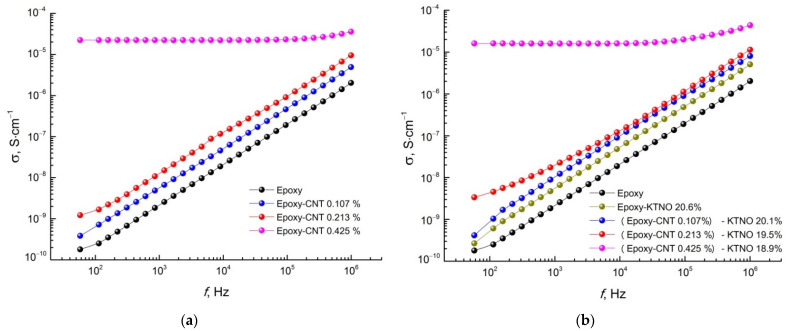
The frequency dependence of the conductivity of (**a**) two- and (**b**) three-phase composites in comparison with the pure epoxy resin.

**Figure 12:**
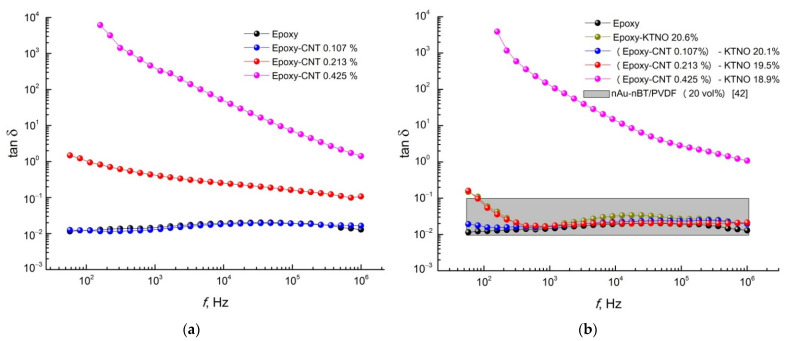
The frequency dependence of the dielectric loss tangent of (**a**) two- and (**b**) three-phase composites in comparison with the pure epoxy resin. Grey section in Figure 12b is experimental data for similar three-phase composite [42].

The dielectric properties of the obtained composites were investigated in comparison with the pure polymer matrix in the frequency range of 100 Hz to 1 MHz at room temperature (Figure 10, Figure 11 and Figure 12).

It was found that the permittivity of the pure polymer matrix in the entire frequency range has a value of about 5. After the addition of carbon nanotubes in an amount of 0.107 vol.% in two-phase composites the value of ε′ increases to 10. A further increase in the concentration of conducting particles in the composition of two-phase composites based on the epoxy resin of 0.213 and 0.425 vol.% leads to an increase in permittivity; this is especially noticeable in low-frequency areas. It should be noted that composites with high CNT concentrations exhibit a frequency-dependent behavior unlike epoxy resin and composite with a low carbon nanotubes content. However, for a composite of Epoxy-CNT 0.213 vol.%, a frequency-independent section is observed in the high-frequency region. In the case of Epoxy-CNT 0.425 vol.%, the frequency-independent section shifts to the region of lower frequencies. The numerical simulation of possible ε′ value in the studied frequency range for two-phase composites with similar compositions from research [41] is plotted in Figure 10a by two different color areas for two different composites. It can be seen that experimental data have good agreement with the simulation results. The introduction of ceramic filler in the amount of 20.6 vol.% into an epoxy resin in the absence of a carbon additive causes an increase in permittivity by several units (from 5 to 14 (inset in Figure 10b)), similar to the three-phase composite of (Epoxy-CNT 0.107%)-KNTO 20.1%. The addition of carbon nanotubes in the amount of 0.213 vol.% to the three-phase composite provides a permittivity from 21.4 to ~23.6 units at 1 MHz and 1 kHz, respectively. In the case of a composite with the highest CNT concentration (0.425 vol.%), a significant increase in ε′ in comparison with the initial polymer matrix and composites with different compositions is associated with an increase in the conductivity of the system after overcoming the percolation threshold, which is also confirmed by the frequency dependences of the conductivity (Figure 11). The obtained permittivity values of (Epoxy-CNT 0.213%)-KNTO 19.5% and (Epoxy-CNT 0.425%)-KNTO 18.9% are comparable to the results for the composite with the composition Au–BaTiO_3_/PVDF (grey section in Figure 10b) [42].

The obtained composites, as well as the pure polymer matrix, are characterized by a similar form of the frequency dependence of conductivity in logarithmic coordinates, which has a rectilinear character, indicating a power law of the dependence of conductivity on frequency. An exception re the epoxy-CNT 0.425% and (Epoxy-CNT 0.425%)-KNTO 18.9% composites, which demonstrate an almost constant conductivity value in the entire investigated frequency range. Therefore, they exhibit the properties of a conductor, which confirms the overcoming of the percolation threshold and the formation of a conductive CNT network.

High values of the permittivity and the conductivity of the composites Epoxy-CNT 0.425% and (Epoxy-CNT 0.425%)-KNTO 18.9% are also accompanied by undesirably high dielectric losses (Figure 12). A small addition of carbon nanotubes (0.107 vol.%), both alone and in combination with a ceramic filler, practically does not affect the dielectric loss tangent of relatively pure epoxy resin, especially at high frequencies. The introduction of K_1.6_(Ni_0.8_Ti_7.2_)O_16_ without a conductive additive and with CNTs in an amount of 0.213 vol.% in the epoxy resin gives the same trend in the frequency dependences of the dielectric loss tangent, exceeding the value of tanδ of ED-20 only in the low-frequency range (~10^2^ Hz). Dielectric losses of the obtained three-phase composites with low CNT content are in the range typical for similar composite Au–BaTiO_3_/PVDF (grey section in Figure 12b) [42].

Of greatest interest is the combination of high permittivity and low dielectric losses. The presence of a synergistic effect when using ceramic and conductive fillers in the production of three-phase polymer-matrix composites is known, which in the study [43] was estimated by the difference in the values of the permittivity of the composite and individual components.

**Figure 13:**
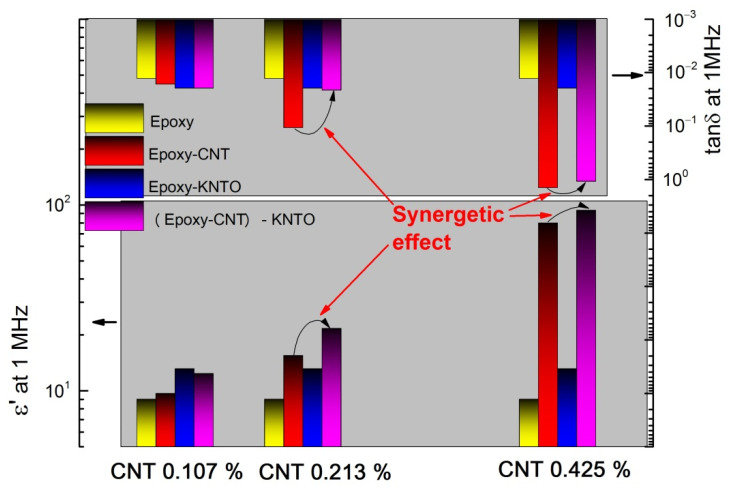
The permittivity and the dielectric loss tangent of pure epoxy resin, two- and three-phase composites with different compositions at 1 MHz.

The synergetic effect in composites obtained in this research is assessed by comparing the change of ε′ and tanδ for the epoxy after adding fillers one by one and together (Figure 13).

In the case of the composite (Epoxy-CNT 0.107%)-KNTO 20.1%, synergy was not observed. This is presumably caused by the lack of contact between the CNT and KNTO particles. On the contrary, the composite (Epoxy-CNT 0.425%)-KNTO 18.9% is characterized by a high concentration of fillers, which is why part of the microcapacitors in this composite are closed. This causes an increase in electrical conductivity and not an accumulation of capacitance, and, as a result, an increase in the permittivity of the composite. In the case of a composite material with a maximum concentration of carbon nanotubes, the synergistic effect is small with the maximum percentage contribution to ε′ from the conductive additive. This can be explained by the formation of a percolation network, accompanied by a change in the insulating and semiconducting properties of the composite to the conductive, which is confirmed by the data on conductivity. The high permittivity of the three-phase composite (Epoxy-CNT 0.213%)-KNTO 19.5% is associated with the formation of a large number of microcapacitors in the form of ceramic dielectric particles of the composition K_1.6_(Ni_0.8_Ti_7.2_)O_16_ with CNTs as electrodes with the existence of an extensive phase boundary between the polymer and fillers, which contributes to the appearance of a better effect of interfacial polarization. Additionally, the decrease in dielectric losses as a positive synergetic effect was observed at concentrations of carbon nanotubes of 0.213 and 0.425 vol.%.

Therefore, based on the foregoing, taking into account the maximum value of the permittivity at relatively low dielectric losses, the optimal solution for three-phase composites based on epoxy resin and ceramic filler K_1.6_(Ni_0.8_Ti_7.2_)O_16_ can be considered as the addition of CNTs in the range of 0.213 vol.% to the percolation threshold.

## 4. Conclusions

Three-phase composites prepared on the basis of epoxy resin (ED-20), a ceramic filler in the form of a complex oxide with a hollandite-like structure of the composition K_1.6_(Ni_0.8_Ti_7.2_)O_16_, and a conductive additive, for which carbon nanotubes were used, were studied by Fourier-transform infrared spectroscopy, scanning electron microscopy and impedance spectroscopy in comparison with the pure polymer matrix and two-phase composites without a ceramic filler. The structure and parameters of the crystal lattice of the oxide material were confirmed and refined using X-ray phase analysis and the Rietveld method. According to the data of scanning electron microscopy, fillers are evenly distributed throughout the entire volume of the polymer matrix. The dielectric properties (permittivity, conductivity, dielectric loss tangent) of composite materials noticeably depend on both the composition and the ratio of the components of the composites and the frequency. With a carbon additive of 0.425 vol.%, two- and three-phase polymer matrix composites show a sharp change in their characteristics, exhibiting the properties of a conductor in contrast to other composites, which is explained by the formation of a continuous conducting CNT network. The minimum concentration of carbon nanotubes (0.107 vol.%) in the composites does not cause a significant increase in dielectric characteristics. The maximum synergistic effect of carbon nanotubes and hollandite K_1.6_(Ni_0.8_Ti_7.2_)O_16_ on the dielectric properties of a composite based on the epoxy resin was found for the composite (Epoxy-CNT 0.213%)-KNTO 19.5%.

## Figures and Tables

**Table 1:** Properties of ED-20 and TETA.

Characteristics	Value
ED-20	
Content of epoxy groups, %	20.0–22.5
Viscosity, Pa∙s	13–20
Epoxy equivalent, g/mol	195–216
Density at 25 °C, kg/m^3^	1166
TETA	
Molecular mass, g/mol	230–250
Viscosity, Pa∙s	0.60–0.90
Density at 25 °C, kg/m^3^	1020
Amine number, mg KOH/g	1250
Nitrogen content, % by weight	30.0

**Table 2:** The used composite components.

Initial Components		Component Content in Composites, vol.%		
Full Name	Short Name			
Epoxy resin	Epoxy			
K_1.6_(Ni_0.8_Ti_7.2_)O_16_	KNTO	20.1	19.5	18.9
Carbon nanotube	CNT	0.107	0.213	0.425

**Table 3:** The obtained and studied composites.

**ED-20**	**KNTO**		**CNT**	
	20.6	Epoxy-KNTO	0.107	(Epoxy-CNT 0.107%)-KNTO 20.1%
			0.213	(Epoxy-CNT 0.213%)-KNTO 19.5%
			0.425	(Epoxy-CNT 0.425%)-KNTO 18.9%
**ED-20**			**CNT**	
			0.107	Epoxy-CNT 0.107%
			0.213	Epoxy-CNT 0.213%
			0.425	Epoxy-CNT 0.425%

## Data Availability

Not applicable.
